# Prevalence of ESBL-producing *Escherichia coli* in adults with and without HIV presenting with urinary tract infections to primary care clinics in Zimbabwe

**DOI:** 10.1093/jacamr/dlab082

**Published:** 2021-06-30

**Authors:** Ioana D Olaru, Rashida A Ferrand, Mutsawashe Chisenga, Shunmay Yeung, Bruce Macrae, Prosper Chonzi, Richard A Stabler, Heidi Hopkins, David Mabey, Kudzai P E Masunda, Katharina Kranzer

**Affiliations:** 1 London School of Hygiene and Tropical Medicine, Keppel Street, London WC1E 7HT, UK; 2 Biomedical Research and Training Institute, 10 Seagrave Road, Harare, Zimbabwe; 3 St Mary’s Imperial College Hospital, Praed Street, Paddington, London W2 1NY, UK; 4 Clinical Microbiology, University College London Hospitals NHS Foundation Trust, 235 Euston Road, Bloomsbury, London NW1 2BU, UK; 5 Department of Health, Harare City Council, Rowan Martin Building, 1 Pennefather Avenue, Harare, Zimbabwe; 6 Division of Infectious and Tropical Medicine, Medical Centre of the University of Munich, Leopoldstrasse, 80802, Munich, Germany

## Abstract

**Background:**

People living with HIV may be at increased risk for infections with resistant organisms. Infections with ESBL-producing organisms are of particular concern because they limit treatment options for severe Gram-negative infections in low-resource settings.

**Objectives:**

To investigate the association between HIV status and urinary tract infections (UTIs) with ESBL-producing *Escherichia coli*.

**Patients and methods:**

Cross-sectional study enrolling adults presenting with UTI symptoms to primary care clinics in Harare, Zimbabwe. Demographic and clinical data were collected during interviews and a urine sample was collected for culture from each participant. Antimicrobial susceptibility testing was performed according to EUCAST recommendations.

**Results:**

Of the 1164 who were enrolled into the study, 783 (64%) were female and 387 (33%) were HIV infected. The median age was 35.8 years. Urine cultures were positive in 338 (29.0%) participants, and the majority of bacterial isolates were *E. coli* (*n *=* *254, 75.2%). The presence of ESBL was confirmed in 49/254 (19.3%) *E. coli*. Participants with HIV had a 2.13 (95% CI 1.05–4.32) higher odds of infection with ESBL-producing *E. coli* than individuals without HIV. Also, the prevalence of resistance to most antimicrobials was higher among participants with HIV.

**Conclusions:**

This study found an association between HIV and ESBL-producing *E. coli* in patients presenting with symptoms suggestive of UTI to primary care in Harare. HIV status should be considered when prescribing empirical antimicrobial treatment.

## Introduction

The increase in antimicrobial resistance (AMR) challenges our ability to effectively treat infections leading to excess morbidity and mortality.^1^ Although AMR has been highlighted as a priority on the global health agenda,^2^ surveillance data on the burden of AMR are lacking from many low- and middle-income countries (LMICs).^3^

In understanding the burden of AMR, specific attention needs to be paid to populations who may be at increased risk for infections with resistant organisms such as people living with HIV. Globally, there are currently 38 million people living with HIV; two-thirds in sub-Saharan Africa.^4^ Although there is evidence of an association between HIV and infection and colonization with resistant organisms, research has mainly focused on Gram-positive pathogens while data for Gram-negative infections are sparse.^5–7^

ESBL-producing Gram-negative organisms are of particular concern in LMICs. ESBLs confer resistance to third-generation cephalosporins, which are the drugs of choice for the treatment of severe Gram-negative infections.^8^ A better understanding of the epidemiology of AMR allows optimization of treatment recommendations with the ultimate aim of improving patient management.

Urine cultures performed in outpatients with symptoms of urinary tract infection (UTI) allow estimation of the burden of community-level Gram-negative resistance. The most frequently isolated organism from urine is *Escherichia coli*,^9^ which is also the main cause of Gram-negative sepsis^10^ and a WHO priority pathogen for AMR surveillance.^3^

This study aimed to investigate the association between HIV status and UTIs with ESBL-producing *E. coli* and to investigate the prevalence of AMR in bacteria causing UTIs among adults presenting to primary healthcare clinics (PHCs) in Harare, Zimbabwe.

## Patients and methods

### Ethics

Ethical approval for the ARGUS study was obtained from the Medical Research Council Zimbabwe (MRCZ/A/2406), the Institutional Review Board of the Biomedical Research and Training Institute in Zimbabwe and the London School of Hygiene and Tropical Medicine Ethics committee (Ref. 16424). The research was conducted in accordance with the Declaration of Helsinki and national and institutional standards. All study participants provided written informed consent.

### Study setting and participants

Data were collected as part of the Antimicrobial Resistance in Gram-negative bacteria from Urinary Specimens (ARGUS) study.^11^ This was a cross-sectional analysis of consecutively enrolled participants recruited from 10 PHCs in southwest Harare between 1 July 2019 and 24 July 2020. Adult HIV prevalence in Zimbabwe is estimated at 13%.^4^ According to national guidelines UTIs should be treated with either a fluoroquinolone or amoxicillin.^12^

Eligibility criteria included age 18 years or older, having at least two symptoms suggestive of UTI, onset of symptoms within the previous 2 weeks, presence of symptoms within the last 24 h, and provision of written informed consent. Those who were discharged from hospital in the previous 72 h, who had a urinary catheter *in situ* or who were enrolled into the study on a previous occasion were excluded.

Interviewer-administered questionnaires determined potential risk factors for AMR and clinical history. Responses were entered in electronic form using the Open Data Kit (ODK, www.opendatakit.org). HIV status was ascertained by self-report and confirmed by patient-held records.

### Laboratory methods

A mid-stream urine sample was collected from every participant and transported to the laboratory within 4 h of collection. A volume of 1 μL of the sample was inoculated on chromogenic agar (Brilliance UTI agar, Oxoid, UK) and incubated at 37 °C for 24 h. Bacterial identification was performed using chromogenic media and APIs (Analytical Profile Index, bioMérieux, France) for Enterobacterales other than *E. coli.* Urine cultures were considered positive if there was growth of ≥10^3^ cfu/mL of a uropathogen either in pure culture or when a uropathogen was predominant. Antimicrobial susceptibility testing (AST) was done by disc diffusion using the Kirby-Bauer method and interpreted using EUCAST standards.^13^ Testing for ESBL and AmpC production was performed according to EUCAST recommendations.^14^ ATCC reference isolates were used for quality control of bacterial identification and AST (for further details on laboratory testing see Supplementary data, available at *JAC-AMR* Online).

The χ^2^ test was used to evaluate categorical variables. Differences between continuous variables were assessed using the Mann–Whitney *U*-test. The level of significance was considered to be *P *≤* *0.05. For the association between HIV infection and infection with ESBL-producing *E. coli*, a multivariate analysis using logistic regression was performed (Figure S1). The model excluded individuals with unknown HIV status. Statistical analyses were performed using STATA v.15 (StataCorp, TX, USA).

### Data availability

All data are presented in the main manuscript and additional supporting files. Data can also be provided on reasonable request to the corresponding author.

## Results

Of the 1374 individuals with UTI symptoms presenting at the clinics, 1164 were eligible and were enrolled into the study. Reasons for ineligibility are detailed in Figure S2.

The median age was 35.8 years (IQR 26.3–47.7) and 743 (63.8%) were female of whom 102 (13.7%) were pregnant (Table 1).

**Table 1.** Characteristics of individuals presenting with symptoms of UTI to public health clinics in Harare, Zimbabwe, stratified by urine culture result

Characteristic	Urine culture result			
	total, *N *=* *1164	positive, *N *=* *338	negative, *N *=* *761	contamination, *N *=* *65
Age, years, median (IQR)	35 (26–48)	34 (25–50)	36 (27–47)	34 (25–47)
Female sex, *n* (%)	743 (63.8)	263 (77.8)	431 (56.6)	49 (75.4)
Pregnancy, *n* (%)^a^				
not pregnant	599 (81.9)	222 (85.4)	341 (80.8)	36 (73.5)
first trimester	19 (1.6)	9 (2.7)	9 (1.2)	1 (1.5)
second trimester	32 (2.7)	8 (2.4)	20 (2.6)	4 (6.2)
third trimester	49 (4.2)	7 (2.1)	35 (4.6)	7 (10.8)
unknown	30 (4.1)	14 (5.4)	15 (3.6)	1 (2.0)
Place of recruitment, *n* (%)				
acute clinic	840 (72.2)	258 (76.3)	539 (70.8)	43 (66.2)
maternity	85 (7.3)	17 (5.0)	56 (7.4)	12 (18.5)
HIV clinic	239 (20.5)	63 (18.6)	166 (21.8)	10 (15.4)
Clinical symptoms				
duration of symptoms, days, median (IQR)	7 (4–12)	7 (3–10)	7 (5–12)	7 (5–12)
reported fever, *n* (%)^b^	122 (10.6)	36 (10.8)	79 (10.5)	7 (10.9)
dysuria, *n* (%)	942 (81.8)	295 (88.1)	595 (79.1)	52 (81.3)
frequency, *n* (%)	824 (71.6)	261 (77.9)	515 (68.5)	48 (75.0)
suprapubic pain, *n* (%)	730 (63.4)	193 (57.6)	490 (65.2)	47 (73.4)
haematuria, *n* (%)	185 (18.8)	77 (23.0)	96 (12.8)	12 (18.8)
limitation of daily activities, *n* (%)	168 (14.6)	60 (17.9)	99 (13.2)	9 (14.1)
Comorbidities, *n* (%)				
diabetes	23 (2.0)	8 (2.4)	13 (1.8)	2 (3.1)
chronic kidney disease	10 (0.9)	3 (0.9)	7 (0.9)	0 (0)
hypertension	126 (11.0)	42 (12.6)	78 (10.5)	6 (9.2)
HIV infection, *n* (%)^c^	387 (36.3)	110 (36.5)	259 (36.8)	18 (29.0)
co-trimoxazole prophylaxis, *n* (%)^d^	214 (55.6)	58 (52.7)	145 (56.4)	11 (61.1)
on ART, *n* (%)^d^	351 (97.2)	99 (97.1)	237 (97.9)	15 (88.2)
Outcome of their clinic visit, *n* (%)				
discharged home	1069 (97.9)	318 (98.8)	691 (97.6)	60 (96.8)
referred to outpatient	12 (1.1)	4 (1.2)	8 (1.1)	0 (0)
specialist				
referred to hospital	11 (1.0)	0 (0)	9 (1.3)	2 (3.2)

Missing data: clinical symptoms (*n *=* *13: duration of symptoms, fever, dysuria, frequency, suprapubic pain, haematuria, limitation of daily activities); comorbidities (*n *=* *23: diabetes, chronic kidney disease, hypertension); ART (*n *=* *26); outcome of the clinic visit (*n *=* *72).

a Denominator: women.

b For 939 (80.7%) participants a temperature measurement was available and 44 (4.7%) had an axillary temperature of ≥37.5 °C.

c 97 patients did not know their HIV status.

d Denominator: participants with HIV infection.

The study included 387 (33.2%) participants with HIV infection, 680 who were HIV negative and 97 (8.3%) who did not know their HIV status. ART coverage among participants with HIV was 97.2% and 214 (55.6%) were receiving co-trimoxazole prophylaxis.

### Uropathogens and AMR

Cultures were positive in 338 (29.0%) participants, 761 (65.4%) were negative and 65 (5.6%) contaminated. The majority of bacterial isolates were *E. coli* (*n *=* *254, 75.2%) followed by other coliforms (*n *=* *33, 9.8%), *Enterococcus* spp. (*n *=* *40, 11.8%), *Staphylococcus saprophyticus* (*n *=* *3, 0.9%) and *Staphylococcus aureus* (*n *=* *2, 0.6%).

The prevalence of resistance to first-line antimicrobials was 245 (79.0%) for amoxicillin and 66 (19.8%) for ciprofloxacin. Co-trimoxazole resistance was present in 247 (84.0%) of isolates. Table S3 shows the prevalence of resistance according to bacterial species. In Enterobacterales resistance to amoxicillin/clavulanic acid was 40.1% (117/292), to nitrofurantoin 9.6% (28/292) and to fosfomycin 2.2% (5/228). Ceftriaxone resistance was present in 52/292 (17.8%) of isolates. The presence of ESBL was confirmed in 49/254 (19.3%) *E. coli*. Exposure to fluoroquinolones in the previous year but not to other antimicrobials was also associated with infections with ESBL-producing *E. coli* (20.4% versus 4.4% for no prior exposure, *P *<* *0.001, Table S5). ESBL-producing *E. coli* were resistant to ciprofloxacin, gentamicin, nitrofurantoin and fosfomycin in 69.4% (34/49), 30.6% (15/49), 18.4% (9/49) and 9.8% (4/41) of isolates, respectively.

### HIV and AMR in E. coli

**Figure 1.**
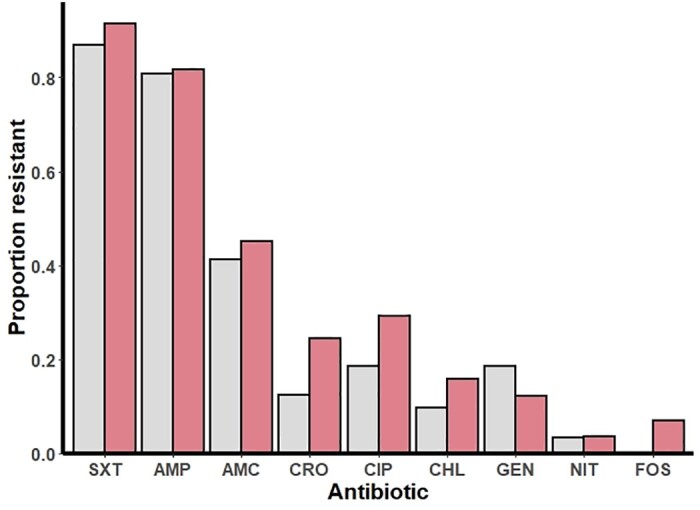
AMR among *E. coli* isolates in individuals with HIV (red) and individuals without HIV infection (light grey). AMP, ampicillin; AMC, amoxicillin/clavulanic acid; CHL, chloramphenicol; CIP, ciprofloxacin; CRO, ceftriaxone; FOS, fosfomycin; GEN, gentamicin; NIT, nitrofurantoin; SXT, co-trimoxazole. None of the isolates had imipenem resistance.

Amoxicillin resistance was present in 67 (81.7%) *E. coli* isolates from participants with HIV and in 117 (80.7%, *P *=* *0.851) from individuals without HIV infection while ciprofloxacin resistance was detected in 24 (29.3%) and 27 (18.6%, *P *=* *0.065), respectively (Figure 1). The prevalence of co-trimoxazole resistance was 91.5% among individuals with HIV and 86.9% in those without HIV infection. In participants with HIV who were taking co-trimoxazole prophylaxis the prevalence of co-trimoxazole resistance was 97.8%. Infections with ESBL-producing organisms were more common among participants with HIV than in participants without HIV infection (26.8% versus 13.1%, *P *=* *0.010). Participants with HIV had a 2.43 (95% CI 1.22–4.83) higher odds of infection with ESBL-producing *E. coli* than individuals without HIV, and the association persisted after adjusting for age and sex (adjusted OR (aOR) 2.13; 95% CI 1.05–4.32, Tables S5 and S6, Figure S1).

## Discussion

This study shows a high prevalence of resistance to first-line antibiotics for UTI treatment and of ESBL-producing *E. coli* among individuals presenting with symptoms suggestive of UTIs to PHCs in Harare. Also, the study found an association between HIV infection and the presence of ESBL-producing *E. coli* in this setting. In general, the prevalence of resistance was higher in participants with HIV infection compared with those without for almost all antimicrobials tested.

Almost one in five of all study participants, and one in four of those with HIV infection, had a UTI due to ESBL-producing *E. coli.* The prevalence of ESBL among participants with HIV was 2-fold higher compared with those without. While resistance to third-generation cephalosporins is less important in primary care settings the associated resistance to oral antimicrobials limits treatment options in outpatients. Furthermore, a high ESBL prevalence in the community setting will translate to high levels of infections due to ESBL-producing organisms among severely ill patients admitted to hospital. This is particularly problematic since third-generation cephalosporins are reserved for the treatment of severe Gram-negative infections in hospitalized patients and alternative treatment options such as carbapenems are not easily available or affordable in most LMICs. This is compounded by limited access to laboratory diagnostics that would enable diagnosis of infections with resistant organisms to guide targeted antibiotic treatment.

Bacterial infections remain a major cause of hospitalization and death among people living with HIV.^15^ The higher prevalence of ESBL-producing *E. coli* infections among participants with HIV may be explained by the more frequent healthcare seeking in this group leading to colonization and infection with resistant organisms. Comorbidities, hospital admissions, clinic visits and antimicrobial prescriptions, which are well-recognized risk factors for infections with resistant pathogens, are more common in those infected with HIV compared with those without.^16–19^ HIV status therefore should be taken into consideration when prescribing antimicrobials.

In this study, there was a high prevalence of resistance to first-line treatment. This is not surprising given the frequent use of amoxicillin for a broad range of indications. Furthermore, the high prevalence of co-trimoxazole resistance can be explained by its use as prophylaxis in HIV-infected individuals.^20^ On the other hand, the prevalence of resistance to nitrofurantoin was <10%, making it an effective and affordable treatment option for uncomplicated infections in this setting at a cost per treatment of approximately $1. Fosfomycin would serve as a good alternative for treatment given the low prevalence of resistance, low cost (around $5), and single-dose treatment regimens.

This study focused on patients presenting to primary care facilities, providing a good estimate for community-level resistance prevalence in Harare. The study recruited from 10 PHCs in the largest city in Zimbabwe across different socioeconomic strata, making the results generalizable to the urban population of Harare. Antimicrobials taken prior to clinic presentation may have led to an overestimation of resistance. Furthermore, the study was underpowered to detect an association between HIV and AMR other than ESBL.

This study found an association between HIV and ESBL-producing *E. coli* in patients presenting with symptoms suggestive of UTI to primary care in Harare. HIV status should be considered when prescribing empirical antimicrobial treatment in this setting to ensure effective treatment for both in- and outpatients. The high prevalence of resistance to first-line antimicrobials highlights the urgent need for up-to-date data on AMR prevalence to inform antimicrobial treatment guidelines. Consolidating laboratory capacity, strengthening AMR surveillance and promoting research in AMR in LMICs is paramount to ensure treatment guidelines are tailored to the local context and epidemiology.

## Funding

This work was supported by funding (to I.D.O.) through the Wellcome Trust Clinical PhD Programme awarded to the London School of Hygiene & Tropical Medicine (grant number 203905/Z/16/Z). The study was funded by UK aid from the UK government.

## Transparency declarations

None to declare.

## Disclaimer

The funders had no role in study design, data collection and interpretation, or the decision to submit the work for publication. The views expressed do not necessarily reflect the UK government’s official policies.

## Supplementary data

Methods, Tables S1 to S6 and Figures S1 to S2 are available as Supplementary data at *JAC-AMR* Online.

## Supplementary Material

dlab082_Supplementary_Data
