# Supplementary material for: Prevalence of ESBL-producing Escherichia coli in adults with and without HIV presenting with urinary tract infections to primary care clinics in Zimbabwe
Source: JAC Antimicrob Resist. 2021 Jun 30;3(2):dlab082. doi: 10.1093/jacamr/dlab082 (PMC8242135; doi:10.1093/jacamr/dlab082)
Supplement: dlab082_Supplementary_Data [file dlab082_supplementary_data.docx]

**Supplementary data**

**TABLE OF CONTENTS**

[**Laboratory methods and sample processing** 2](#_Toc70843931)

[**Table S1** Antimicrobial susceptibility testing according to pathogen 2](#_Toc70843932)

[**Sample size and statistical analysis** 3](#_Toc70843933)

[**Figure S1** Directed acyclic graph for the relationship between HIV infection (exposure) and infection with ESBL-*E. coli* (outcome) 4](#_Toc70843934)

[**Figure S2.** Flow diagram of patients included in the study 5](#_Toc70843935)

[**Urine dipstick results** 5](#_Toc70843936)

[**Table S2.** Bacterial growth in urine cultures and urine dipstick results according to HIV status. 6](#_Toc70843937)

[**Prevalence of resistance** 6](#_Toc70843938)

[**Table S3.** Antimicrobial resistance according to bacterial species isolated 6](#_Toc70843939)

[**Table S4.** Prevalence of resistance in *E. coli*. 7](#_Toc70843940)

[**Risk factor analysis and the association between HIV and infections with ESBL-*E. coli*** 7](#_Toc70843941)

[**Table S5.** Characteristics of individuals with *E. coli* UTIs, stratified by presence of ESBL 7](#_Toc70843942)

[**Table S6.** Univariate and multivariate analysis of the association between HIV and urinary tract infection with ESBL-*E. coli* 8](#_Toc70843943)

# **Laboratory methods and sample processing**

Urine samples underwent dipstick, microscopy and culture on the day of collection. Urine dipstick was considered positive if either nitrites or leucocytes were present. Leukocyturia was considered to be present if the sample had ≥10 white blood cells/µl on microscopy.

A volume of 1µL of the sample was inoculated on chromogenic agar (Brilliance UTI agar, Oxoid, UK) and incubated at 37°C for 24 hours. Urine cultures were considered positive if there was growth of ≥10^3^ colony forming units (cfu)/mL. This threshold for positivity was used given that lower bacterial counts can be significant in patients with UTI symptoms.[13] Cultures were considered contaminated if growth of a non-uropathogen was present or if ≥2 organisms were isolated in the absence of a clear predominance of one organism. To minimise contamination, samples were refrigerated if a prolonged transportation time was anticipated.

*E. coli* was identified by colony appearance on chromogenic media while other *Enterobacterales* were identified using APIs (Analytical Profile Index, bioMérieux, France). This strategy may have led to the misidentification of a small number of *Citrobacter spp.,* as *E. coli.* However, it is anticipated that misidentification was infrequent and it would not have impacted the overall study results. The identity of presumptive *E. coli* isolates showing discoloration on chromogenic media was confirmed using APIs.

Drugs used for AST are shown in Table S1. Fosfomycin AST was done for *E. coli* using disc diffusion testing. Fosfomycin AST for other *Enterobacterales* requires determination of minimum inhibitory concentrations which was not available.

## **Table S1** Antimicrobial susceptibility testing according to pathogen

| **Organism** | **Drugs tests for AST^#^** |
| --- | --- |
| *E. coli* | Ampicillin, Amoxicillin/ clavulanic acid, cefpodoxime, ceftazidime, ceftriaxone, cefoxitin, imipenem, ciprofloxacin, amikacin, gentamicin, co-trimoxazole, chloramphenicol, nitrofurantoin, fosfomycin |
| Other *Enterobacterales* | Ampicillin, Amoxicillin/ clavulanic acid, cefpodoxime, ceftazidime, ceftriaxone, cefoxitin, imipenem, ciprofloxacin, gentamicin, co-trimoxazole, chloramphenicol, nitrofurantoin |
| *Enterococcus spp.* | Ampicillin, ciprofloxacin, nitrofurantoin |
| *Staphylococcus saprophyticus* | Cefoxitin, ciprofloxacin, nitrofurantoin, co-trimoxazole |

*^#^Internal quality control for AST was conducted and interpreted in accordance to EUCAST recommendations (Routine and extended internal quality control for MIC determination and disk diffusion as recommended by EUCAST Version 9.0, valid from 2019-01-01, www.eucast.org)*

Testing for ESBL and AmpC production was performed according to EUCAST recommendations.[15] Briefly, screening for the presence of ESBLs was done using cefpodoxime. Isolates positive on the screening test underwent confirmation by synergy testing with amoxicillin/clavulanic acid and ceftazidime. Screening for AmpC was performed using cefoxitin and ceftazidime and was confirmed by cloxacillin synergy testing.[15]

Quality of laboratory testing was ensured by using standard operating procedures, training and regular re-training on laboratory procedures and supervision by a senior clinical microbiologist.

# **Sample size and statistical analysis**

Sample size calculations were conducted for the association between HIV status and infection with ESBL-*E. coli*. The following was assumed: i) 30% of urine cultures positive, ii) 90% of organism *E. coli*, iii) 25% of participants infected with HIV, 15% of ESBL-*E. coli* among HIV uninfected and 30% among HIV-infected participants. A sample size of 1404 would detect a difference with 80% power and a level of significance of 5%.

For the association between HIV infection and infection with *ESBL*-*E. coli*, a multivariate analysis using logistic regression was performed. Directed acyclical graphs were used to establish variables on the causal pathway between the exposure and the outcome and these were not adjusted for in the multivariate model (Figure S1. Variables that were associated with the exposure of interest (HIV infection) and with the outcome (infection with ESBL-*E. coli*) were included in the multivariate model.

## **Figure S1** Directed acyclic graph for the relationship between HIV infection (exposure) and infection with ESBL-*E. coli* (outcome). Variables in red are potential confounders while the green arrows show a causal path. Variables in blue are ancestors of the outcome.


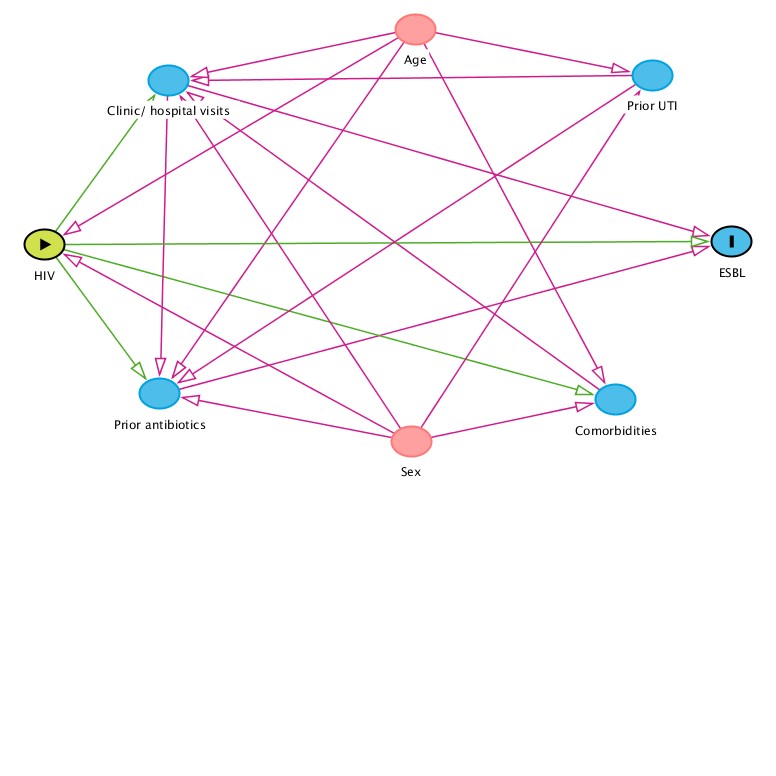


*Clinic/ hospital visits, prior antibiotics (including co-trimoxazole prophylaxis) and comorbidities are on the causal pathway between the exposure of interest (HIV infection) and the outcome (infection with ESBL-producing organisms). These variables cannot be considered confounders of the association. The diagram was created using www.dagitty.net.*

# **Figure S2.** Flow diagram of patients included in the study


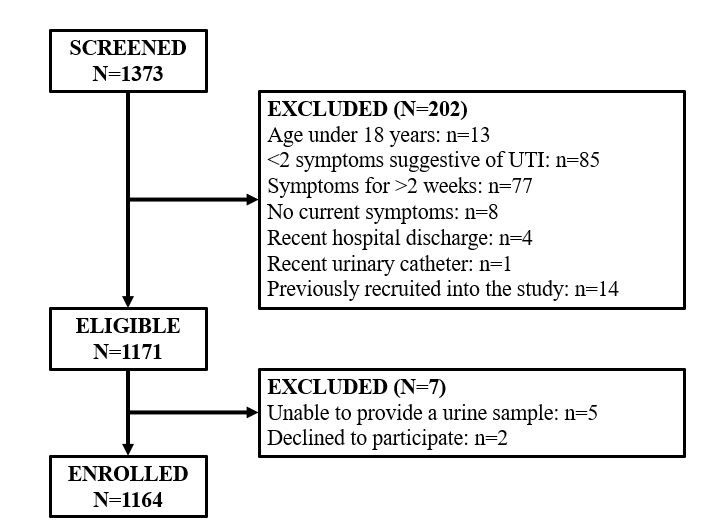


*The study was unable to recruit to the planned sample size because of suspension of research activities during the national COVID-19 lockdown (April-May 2020) and because recruitment had to be stopped earlier than anticipated (July 2020) due to COVID-19-related clinic closures.*

# **Urine dipstick results**

A positive urine dipstick was recorded in 411 (35.4%) and 321 (27.6%) had positive urine microscopy for leucocytes. Overall, the urinary dipstick had a sensitivity of 71% (95% CI 66-76) and a specificity of 80% (95%CI 77-83) for predicting a positive urine culture (Table S2). There was a good concordance between microscopy and dipstick leucocyte esterase results of 88.9% (1033/1162).

## **Table S2.** Bacterial growth in urine cultures and urine dipstick results according to HIV status.

| **Patient category** | **Leucocyte esterase positive** | **Nitrite positive** | **Combined leucocyte esterase or nitrite positive** | |
| --- | --- | --- | --- | --- |
|  | **N (%)** | **N (%)** |  | |
|  |  |  | **Sensitivity** | **Specificity** |
| ***HIV negative (n=678)*** |  |  | 69% (62-76) | 81% (77-84) |
| Culture results |  |  |  |  |
| *Enterobacterales* (n=169) | 123 (73) | 72 (43) |  |  |
| Gram-positive bacteria (n=23) | 8 (35) | 0 (0) |  |  |
| Contamination (n=42) | 14 (32) | 1 (2) |  |  |
| Culture negative (n=444) | 84 (19) | 6 (1) |  |  |
| ***HIV positive (n=387)*** |  |  | 68% (59-77) | 81% (76-86) |
| Culture results |  |  |  |  |
| *Enterobacterales* (n=91) | 69 (76) | 34 (37) |  |  |
| Gram-positive bacteria (n=19) | 5 (26) | 0 (0) |  |  |
| Contamination (n=18) | 5 (28) | 2 (11) |  |  |
| Culture negative (n=259) | 50 (19) | 4 (2) |  |  |

*Dipstick could not be performed in 2 patients because of sample spillage during transportation. For the sensitivity and specificity calculations, contaminated cultures were excluded.*

# **Prevalence of resistance**

The prevalence of resistance among the isolated bacterial species are shown in Table S3. For *E. coli* prevalence with 95% CIs are shown in Table S4.

## **Table S3.** Antimicrobial resistance according to bacterial species isolated from urine samples from individuals presenting with symptoms of urinary tract infection to public health clinics in Harare, Zimbabwe (percentages are shown in brackets)

|  | **AMP** | **AMC** | **CRO** | **IMP** | **CIP** | **GENT** | **NIT** | **FOS** | **SXT** | **CHL** |
| --- | --- | --- | --- | --- | --- | --- | --- | --- | --- | --- |
| *E. coli* (n=254) | 209 (82) | 104 (41) | 46 (18) | 0 (0) | 57 (22) | 42 (17) | 14 (6) | 5 (2) | 223 (88) | 28 (11) |
| Other *Enterobacterales (n=39)* | - | 13 (34) | 6 (16) | 0 (0) | 2 (5) | 4 (11) | 14 (37) | - | 23 (61) | 4 (11) |
| *Enterococcus spp.* (n=40) | 5 (28) | - | - | - | 7 (18) | - | 4 (10) | - | - | - |

*AMP: ampicillin; AMC: amoxicillin/ clavulanic acid; CRO: ceftriaxone; IMP: imipenem; CIP: ciprofloxacin; GENT: gentamicin; NIT: nitrofurantoin; FOS: fosfomycin; SXT: co-trimoxazole; CHL: chloramphenicol. Other Enterobacterales were: Klebsiella pneumoniae (n=20); Enterobacter spp. (n=6); Proteus mirabilis (n=6); Citrobacter spp. (n=2); Klebsiella oxytoca (n=1); not identified (n=4 – these showed growth of blue colonies on the Brilliance UTI agar). Missing: AST not done (n=2, K. pneumoniae and Enterococcus spp.); AST for ciprofloxacin missing (n=1, E. aerogenes); AST for ampicillin missing (n=21, Enterococcus spp.); AST for Fosfomycin missing (n=30, E. coli). S. saprophyticus (n=3) and S. aureus (n=2) AST not included.*

## **Table S4.** Prevalence of resistance in *E. coli* with 95% confidence intervals.

| **Antibiotic** | **Percentage resistant (95%CI)** |
| --- | --- |
| Amoxicillin | 82% (77-87) |
| Amoxicillin/clavulanic acid | 41% (35-47) |
| Ceftriaxone | 18% (14-23) |
| Imipenem | 0% (0-1) |
| Ciprofloxacin | 22% (17-28) |
| Gentamicin | 17% (12-22) |
| Amikacin | 0% (0-2) |
| Co-trimoxazole | 88% (83-92) |
| Chloramphenicol | 11% (7-16) |
| Nitrofurantoin | 6% (3-9) |
| Fosfomycin | 2% (1-5) |

*AST is shown for all E. coli isolates (N=254) except for amikacin and fosfomycin where n=228 were tested.*

# **Risk factor analysis and the association between HIV and infections with ESBL-*E. coli***

Characteristics of study participants with and without ESBL*-E. coli* infections are shown in Table S5. The univariate and multivariate analyses of the association between HIV and infection with ESBL-*E. coli* are shown in Table S6.

## **Table S5.** Characteristics of individuals presenting to public health clinics in Harare, Zimbabwe, with *E. coli* UTIs, stratified by presence of ESBL

| **Risk factors for resistance** | **ESBL*-E. coli*** | **Non-ESBL *E. coli*** | **p-value** |
| --- | --- | --- | --- |
|  | **N=49** | **N=205** |  |
| Age (years), median (IQR) | 37.3 (30.6-60.4) | 31.8 (24.8-46.7) | 0.016 |
| Female sex | 36 (73.5) | 171 (83.4) | 0.107 |
| Pregnancy | 3 (8.6) | 17 (10.9) | 0.685 |
| HIV infection | 22 (53.6) | 60 (32.3) | 0.010 |
| Prior UTI | 14 (30.4) | 35 (17.4) | 0.046 |
| ***Prior antibiotic use (last 12 months)*** |  |  |  |
| Any antibiotic | 21 (44.7) | 65 (32.3) | 0.109 |
| Amoxicillin | 11 (22.5) | 34 (16.6) | 0.334 |
| Fluoroquinolone | 10 (20.4) | 9 (4.4) | <0.001 |
| Co-trimoxazole (incl. prophylaxis) | 11 (22.5) | 38 (18.5) | 0.533 |
| Prior hospital admission (last 12 months) | 3 (6.4) | 15 (7.5) | 0.797 |

*UTI: urinary tract infection. Missing: unknown HIV status (n=27); prior UTI (n=7); pregnancy status (n=16); age (n=1); previous hospital admission (n=6).*

## **Table S6.** Univariate and multivariate analysis of the association between HIV and urinary tract infection with ESBL-*E. coli*

| **Risk factors for resistance** | **Unadjusted OR (95%CI)** | **p-value** | **Adjusted OR** (95% CI)** | **p-value** |
| --- | --- | --- | --- | --- |
| Age* | 1.02 (1.00-1.04) | 0.030 | 1.02 (0.99-1.04) | 0.128 |
| Female sex | 0.80 (0.34-1.91) | 0.621 | 1.16 (0.45-3.02) | 0.755 |
| HIV infection | 2.43 (1.22-4.83) | 0.011 | 2.13 (1.05-4.32) | 0.036 |

**Age was included as a linear variable. There was no departure from a linear trend for the age variable. **The OR was adjusted for age and sex.*
